# Poly-arginine R18 and R18D (D-enantiomer) peptides reduce infarct volume and improves behavioural outcomes following perinatal hypoxic-ischaemic encephalopathy in the P7 rat

**DOI:** 10.1186/s13041-018-0352-0

**Published:** 2018-02-09

**Authors:** Adam B. Edwards, Jane L. Cross, Ryan S. Anderton, Neville W. Knuckey, Bruno P. Meloni

**Affiliations:** 1 Perron Institute for Neurological and Translational Sciences, QEII Medical Centre, RR Block, Ground Floor, 8 Verdun St, Nedlands, WA 6009 Australia; 2 School of Health Sciences and Institute for Health Research, The University of Notre Dame Australia, Fremantle, WA 6160 Australia; 3 Department of Neurosurgery, Sir Charles Gardiner Hospital, QEII Medical Centre, Western Nedlands, 6009 Australia; 4 Centre for Neuromuscular and Neurological Disorders, The University of Western Australia, Nedlands, WA 6009 Australia

**Keywords:** Hypoxic-ischaemic encephalopathy, Hypoxia-ischaemia, Neuroprotection, Poly-arginine peptides, R18, Cationic arginine-rich peptides (CARPs)

## Abstract

We examined the neuroprotective efficacy of the poly-arginine peptide R18 and its D-enantiomer R18D in a perinatal hypoxic-ischaemic (HI) model in P7 Sprague-Dawley rats. R18 and R18D peptides were administered intraperitoneally at doses of 30, 100, 300 or 1000 nmol/kg immediately after HI (8% O_2_/92%N_2_ for 2.5 h). The previously characterised neuroprotective JNKI-1-TATD peptide at a dose of 1000 nmol/kg was used as a control. Infarct volume and behavioural outcomes were measured 48 h after HI. For the R18 and R18D doses examined, total infarct volume was reduced by 25.93% to 43.80% (*P* = 0.038 to < 0.001). By comparison, the JNKI-1-TATD reduced lesion volume by 25.27% (*P* = 0.073). Moreover, R18 and R18D treatment resulted in significant improvements in behavioural outcomes, while with JNKI-1-TATD there was a trend towards improvement. As an insight into the likely mechanism underlying the effects of R18, R18D and JNKI-1-TATD, the peptides were added to cortical neuronal cultures exposed to glutamic acid excitotoxicity, resulting in up to 89, 100 and 71% neuroprotection, respectively, and a dose dependent inhibition of neuronal calcium influx. The study further confirms the neuroprotective properties of poly-arginine peptides, and suggests a potential therapeutic role for R18 and R18D in the treatment of HIE.

**Electronic supplementary material:**

The online version of this article (10.1186/s13041-018-0352-0) contains supplementary material, which is available to authorized users.

## Introduction

In the human neonate, hypoxic-ischaemic encephalopathy (HIE) remains the leading cause of neonatal mortality and morbidity, with a reported incidence of 1–3 per 1000 live term births [1], [2]. Neurological sequelae associated with HIE include cerebral palsy, epilepsy, mental retardation and learning disabilities [3]. Clinically, treatment to reduce brain injury for HIE is extremely limited, consisting of moderate hypothermia (32–34 °C), maintained for up to 72 h. While hypothermia appears to be well tolerated, its safety has only been assessed in full-term infants (37–40 weeks gestation), hence no treatments are available for preterm neonates with HIE born before 37 weeks gestation. In addition, while hypothermia has been shown to improve outcomes in clinical trials, 31–55% of infants do not benefit from the therapy and suffer severe neurological sequelae [4]. Furthermore, hypothermia can only be used in hospital settings that have the necessary equipment and trained staff to induce and monitor hypothermia following HIE, and thus is not available in non-tertiary hospitals or hospitals located in remote locations and in many developing countries.

Due to the need for additional neuroprotective strategies for HIE, research has focused on the development of a pharmacological neuroprotective agent that can be administered alone and/or in combination with current hypothermia treatment to improve neonatal outcomes in HIE. An added advantage of a pharmacological neuroprotective agent is the possibility it can be used in remote locations, is effective in preterm infants, and/or can improve neuroprotective outcomes for hypothermia. We and others have recently demonstrated that cationic arginine-rich and poly-arginine peptides (hereafter referred to as CARPs) have potent neuroprotective properties in in vitro excitotoxicity neuronal injury models [5–13], as well as in animal stroke models [10, 11, 14–16]. In particular, we have demonstrated that the poly-arginine peptide, R18, is highly neuroprotective in permanent and transient middle cerebral artery occlusion (MCAO) stroke models [11, 14–16].

While the exact mechanisms of the neuroprotective effects are still being explored, we and others have demonstrated that CARPs, including “putative” neuroprotective peptides fused to cationic arginine-rich cell penetrating peptides (i.e. TAT or R9; e.g. JNKI-1-TATD, TAT-NR2B9c/NA-1, TAT-CBD3/R9-CBD3-A6K and TAT-3.2-III-IV) have the ability to mitigate excitotoxic or potassium evoked neuronal intracellular calcium influx [7, 8, 17–19], and in doing so, likely suppress damaging down-stream signaling and cell death pathways. Beyond this, we have demonstrated that the poly-arginine R12 and TAT-NR2B9c/NA-1 peptides reduce neuronal cell surface expression of the glutamate receptor subunit NR2B [20]. The ability of CARPs to reduce neuronal cell surface levels of NR2B (due to internalization during endocytic uptake) is at least one mechanism whereby the peptides reduce the damaging effects of glutamate-induced calcium influx and excitotoxicity. Additionally, other “putative” neuroprotective peptides bound to TAT or R9 have been shown to reduce neuronal surface expression or activity of NMDA receptors [5, 21–23], as well as voltage gated calcium channels CaV2.2 [21, 24, 25] and CaV3.3 [18], and the sodium calcium exchanger 3 (NCX3) [21]. Given their effects on different receptors, presently, it cannot be ruled out that CARPs, due to their cationic charge, are also antagonizing ion channel receptor function directly and/or affecting calcium ion influx via an electrostatic mechanism [7, 9].

In addition to CARPs reducing the effects of excitotoxicity, based on other properties of this class of peptide, they may exert more pleiotropic neuroprotective effects by targeting mitochondria and maintaining mitochondrial integrity [6, 26–32], thereby reducing reactive oxygen species (ROS) production and release of pro-cell death proteins such as cytochrome-c. Furthermore, CARPs have the capacity to inhibit proteolytic enzymes including the proteasome [33, 34], as well as proprotein convertases [35–37] that activate matrix metalloproteinases (MMPs) [38]. Inhibition of the proteasome is known to be beneficial following cerebral ischaemia [39, 40], a mechanism that could be mediated by increased activity of hypoxia-inducible factor 1-α (HIF1α) and decreased activity of nuclear factor κ-light-chain-enhancer of B cells (NFκB). Similarly, due to the damaging effects of MMPs on the neurovascular unit and blood brain barrier, any down-regulation of their proteolytic activity is likely to be beneficial following brain HI. In addition, there is also evidence that CARPs can activate pro-survival signaling pathways [17, 41] and modulate immune responses [42–44], as well as act as anti-oxidant molecules in their own right due to their guanidino group containing arginine residues [32, 45–49].

Since HIE and stroke are thought to share many pathophysiological processes (e.g. excitotoxicity and oxidative stress) it is likely that R18 will also be effective in reducing HI brain injury. To this end, several CARPs, including “putative” neuroprotective peptides fused to TAT such as JNKI-1-TATD [50], P5-TAT [51], TAT-NDB [52], TAT-mGluR1 [53], TAT-NR2B9c/NA-1 [53], IDR-1018 [54], and COG133 [55], have demonstrated efficacy in perinatal HI models. Importantly, CARPs like the TAT peptide also have cell penetrating properties and can cross the blood brain barrier and enter the brain [56–58].

Therefore, given the demonstrated efficacy of the R18 poly-arginine peptide in a number of experimental stroke studies [11, 14–16], the present study examined the neuroprotective efficacy and dose responsiveness of R18, as well as its D-amino acid enantiomer (R18D) in a perinatal HI model in the P7 Sprague-Dawley rat, and their effects on neuronal calcium influx in an in vitro glutamic acid excitotoxicity model. In addition, as a control, the study also examined the effects of the cationic arginine-rich JNKI-1-TATD peptide, which has previously been shown to be neuroprotective in various rodent brain injury models, including perinatal HI [50, 59].

## Methods

### Animal ethics and study design

All experimental procedures in this study adhered to the guidelines approved and specified by the Animal Ethics Committee of the University of Western Australia (RA/3/100/1329), in accordance with the Policies and Guidelines of the National Health and Medical Research Council, Australia. Treatments were randomised and all procedures (e.g. peptide administration, behavioural testing and infarct volume analysis) were performed while being blinded to treatments.

### Peptides used in the study

Peptides used in the study are shown in Table 1. R18, R18D and JNKI-1-TATD used were synthesised by Mimotopes (Melbourne, Australia). Peptides were purified using high performance liquid chromatography to at least 98% purity, and were subject to peptide hydrolysis and amino acid liquid chromatography analysis to obtain a precise measure of peptide content.

**Table 1:** Peptides used in study

Peptide	Sequence^a^	Arginine/AA residues	% Arginine residues	Net charge
R18	H-RRRRRRRRRRRRRRRRRR-OH	18/18	100	+ 18
R18D	H-rrrrrrrrrrrrrrrrrr-OH	18/18	100	+ 18
JNKD	H-tdqsrpvqpflnlttprkprpp-rrrqrrkkrg-NH_2_	9/32	28	+ 12

^a^At the N-terminus, H indicates free amine. At the C-terminus, OH indicates free acid, and NH_2_ indicates amide. AA = amino acids. Lowercase single letter code indicates D-isoform of the amino acid. JNKD = JNKI-1-TATD

For the in vitro and animal studies, peptides were prepared as a 500 μM stock solution in water for irrigation (Baxter, Australia), and in 0.9% sodium chloride for injection (Pfizer, Australia), respectively. Reconstituted peptides were stored at − 20 °C until use.

### Surgical procedure for modified Rice-Vannucci model

We used a modified Rice-Vannucci HI model. This involved occluding the right common, as well as the right external carotid artery, a procedure that we have previously demonstrated to lead to the generation of a highly reliable infarct [60]. Briefly, unsexed, Sprague-Dawley P7 rat pups (Animal Resource Centre, Murdoch, Australia; P0 = day of birth) with a body weight of 13–16 g were used. Litters were culled to a maximum of 12 pups per litter to facilitate uniform growth without littermate competition. Rat pups were anaesthetised using isoflurane (5% induction, 1–2% maintenance) in 100% oxygen, while on a heating pad (37 °C). Through a 1 cm mid-line ventral incision, the right common, internal and external carotid arteries were exposed and isolated from the vagus nerve, venous circulation and carotid body. The right common carotid and external carotid arteries were permanently ligated using 6–0 silk sutures. The wound was closed using Vetbond (3M, Maplewood, USA) and the animals were recovered on 100% oxygen for 5 min on a heating pad. The duration of anaesthesia from induction to beginning of recovery was between 5 and 8 min. Animals were provided with analgaesia (pethidine, 5 mg/kg; intraperitoneally) immediately before cessation of anaesthesia. Sham-operated animals underwent the same operative procedure, except the exposed carotid arteries were not ligated.

Rat pups were returned to their dam for 1 h before the commencement of hypoxia which consisted of placing 4 to 6 pups in an airtight container (approximate volume: 4 L) and exposing them to humidified and warmed hypoxic gas (8% O_2_/92% N_2_; 3 L/min) for 2.5 h. The container was placed inside an incubator with an ambient air temperature of 35 °C, ensuring a body temperature of 36–37 °C, which was periodically monitored using an infrared thermometer. Following hypoxia, animals were placed on a heating pad for 5 min in a normoxic environment before being placed back with the dam. Sham-operated animals remained with the dam at all times.

### Peptide administration

Immediately after hypoxia, treatments were administered intraperitoneally (50 μl bolus) and consisted of either the vehicle (saline; 0.9% sodium chloride), or R18 or R18D at four different doses (30, 100, 300 or 1000 nmol/kg) or JNKI-1-TATD at a single dose (1000 nmol/kg).

### Animals used and sample size

**Fig. 1:**
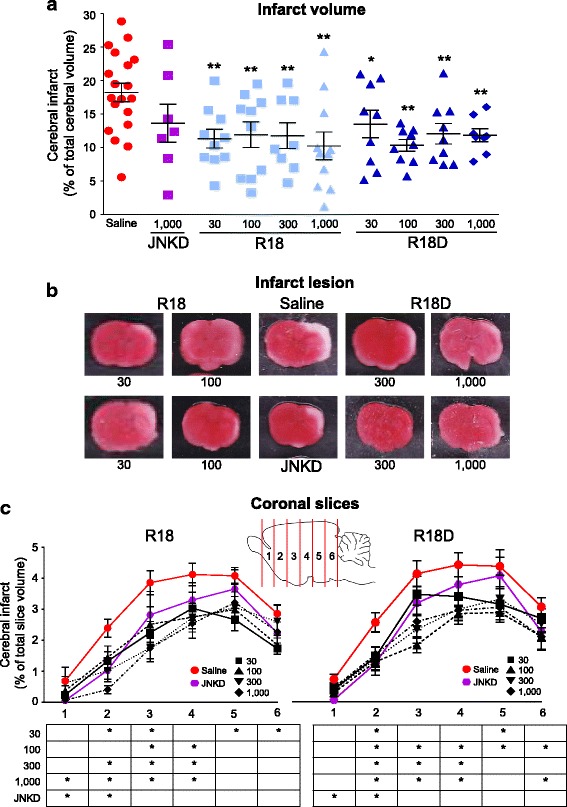
Percentage infarct volume; percentage infarct volume, representative images of coronal brain slices and percentage infarct volume in brain slices for the different treatment groups as determined 48 h after HI. Treatments were administered intraperitoneally (saline or R18, R18D and JNKI-1-TATD; doses in nmol/kg) immediately after hypoxia. **a** Percentage of infarct volume when compared to total brain volume. **b** Representative TTC coronal brain slice 2, from saline and peptide treated animals. Minor adjustments to brightness and contrast has been made to improve digital images. **c** Infarct volume analysis in 2 mm coronal brain slices (slices numbered 1–6 from rostral to caudal) from saline and peptide treated animals. Statistical significance is expressed in the table. Values are mean ± SE; *P < 0.05, **P < 0.01 when compared to saline. JNKD = JNKI-1-TATD

One hundred and thirty-three Sprague-Dawley P7 pups housed under controlled conditions on a 12-h light-dark cycle underwent surgery for HI or the sham procedure (i.e. anesthesia, neck incision, wound closure and analgesia). Twenty-seven animals were excluded from the study; a list of animal exclusions is provided in Table 2. The saline treatment group consisted of 19 animals and the sham group 6 animals. All other treatment groups consisted of between 7 and 11 animals (see Fig. 1a and Table 3). Additional vehicle treated animals were included in the study to ensure that each batch of animals subjected to HI contained a vehicle control and to increase the statistical power of the study.

**Table 2:** Animals excluded in the study

Exclusion rationale	Number of exclusions	Peptide	Dose	Total number of exclusions
No detectable infarct	3	Saline	–	15
	1	R18	300	
	3	R18	100	
	2	R18	300	
	1	R18	1000	
	2	R18D	30	
	1	R18D	300	
	1	R18D	1000	
	1	JNKD	1000	
Hyperthermia^a^	1	R18	1000	1
Failure to complete behavioural assessment	3	–	–	3
Surgical haemorrhage^b^	5	–	–	5
Premature death^c^	3	–	–	3

All peptide doses are in nmol/kg. ^a^Hyperthermia is a recorded body temperature > 37.5. ^b^Surgical haemorrhage is defined as any animal which had an abnormal bleed (arterial rupture) during the surgical parameter. ^c^Premature deaths occurred during hypoxia. JNKD = JNKI-1-TATD

**Table 3:** Cerebral infarct. Percentage of total cerebral volume

Treatment	Dose	N	Mean of total infarct volume (%)	SE (%)	Reduction in total infarct volume (%)	*P*
Saline	–	19	18.24	1.393	–	–
JNKD	1000	7	13.63	2.843	25.27	0.073
R18	30	10	11.34	1.401	37.82	**0.002**
	100	10	11.93	1.924	34.59	**0.004**
	300	8	11.78	1.921	35.42	**0.007**
	1000	11	10.25	2.082	43.80	**< 0.001**
R18D	30	9	13.51	2.058	25.93	**0.038**
	100	9	10.33	0.874	43.36	**< 0.001**
	300	9	12.07	1.540	33.82	**0.007**
	1000	8	11.83	0.957	35.14	**0.007**

*N* number of animals, *SE* standard error of mean, *P* calculated compared to saline vehicle control. All doses are in nmol/kg. Mean and SE expressed as percentage of total cerebral volume. JNKD = JNKI-1-TATD. Percentage reduction is mean percentage reduction compared to saline. All values *P* < 0.05 are in bold

### Infarct volume assessment

Forty-eight hours after HI, animals were euthanised by pentobarbital overdose (50 mg/kg; intraperitoneally). Infarct volume was determined by preparing 2 mm thick coronal brain slices, and incubating in 3% 2,3,5-triphenyltetrazolium chloride (TTC; Sigma Aldrich, St. Louis, USA) at 37 °C for 20 min, followed by fixation in 4% formalin at room temperature overnight. Digital images of coronal slices were acquired using a colour scanner and analysed, using ImageJ software (3rd edition, NIH, Bethesda, USA). Total infarct volume was determined by measuring areas of infarcted tissue on both sides of the 2 mm slices. Infarct volume measurements were corrected for a degree of hemisphere volume changes [60]. Final infarct data is expressed as percentage infarct volume compared to whole brain (minus cerebellum). For analysis of infarct volumes in male and female animals, due to low numbers of each gender per group, data for the different doses of R18 and R18D were combined.

### Behavioural assessment

To determine if the development of cerebral infarct was associated with reduced sensorimotor outcomes, three neurological tests (righting reflex, negative geotactic response and wire-hang) were performed at 48 h after HI, as previously reported [60]. Briefly, for three consecutive days before surgery (P4 to P6 inclusive), pups were accustomed to behavioural assessments with a target inclusion range of ≤2 s for the righting reflex and ≤30 s for the negative geotactic response. Any animal that did not record responses within these parameters at P7 (day of surgery) were excluded from the experiment. All of these reflexes are highly reproducible throughout the murine pre-weaning period (< P30) and are strain- and gender-independent [61]. Each animal was given three attempts to complete each sensorimotor task, with 5 min between each attempt. Testing of all reflexes was performed on a board covered with a tightly-stretched close knit-fabric, to ensure adequate friction.

The righting reflex involved placing pups in a supine position, and measuring the time required to rotate to the prone position. The negative geotactic response involved placing pups facing down-slope, on a 45° angled surface, and measuring the time required for the animal to turn 150° upslope. Wire-hang test was performed by suspending pups by their forelimbs on a 2 mm diameter steel wire suspended 20 cm above a foam surface, and recording the time taken for the animal to fall to the foam surface.

### Cortical neuronal cultures

Establishment of rat primary cortical neuronal cultures was performed as previously described [8, 10]. Briefly, cortical tissue was obtained directly from E18-day old embryos. Dissociated neurons were seeded into 96-well-sized glass wells (7 mm diameter, Grace, Melbourne, Australia), 96-well plastic plates (Nunc, Thermo Fisher Scientific, Melbourne, Australia) in Neurobasal/2% B27 supplement (Life Technologies, Melbourne, Australia) and maintained in a CO_2_ incubator (5% CO_2_, 95% air balance, 98% humidity) at 37 °C until use on day in vitro 10 to 14. Under these conditions, cultures routinely consist of > 97% neurons and 1–3% astrocytes.

### Glutamic acid excitotoxicity

A dose response experiment was performed as previously described [8]. Briefly, peptides in a 50 μl volume in Minimum Essential Medium (Life Technologies)/2% B27 supplement (MEM/B27) were added to culture wells for 10 min at 37 °C in the CO_2_ incubator before the addition of 50 μl MEM/B27 containing glutamic acid (_L_-glutamic acid; Sigma-Aldrich: 200 μM; final concentration 100 μM). Following a 5 min incubation at 37 °C in the CO_2_ incubator (note: peptide concentration reduced by half during this step), media was replaced with 100 μL MEM/B27 and cultures wells were incubated for a further 24 h at 37 °C in the CO_2_ incubator. Untreated controls with or without glutamic acid treatment underwent the same incubation steps and media additions.

At different times after treatment (e.g. 0.5 to 4 h and 18–24 h), cultures were examined by light microscopy for qualitative assessment of neuronal cell viability. Neuronal viability was quantitatively measured by MTS (3-(4,5,dimethyliazol-2-yl)-5-(3-carboxymethoxy-phenyl)-2-(4-sulfophenyl)-2H–tetrazolium salt) assay (Promega, Sydney, Australia). The MTS absorbance data were converted to reflect proportional cell viability relative to both the untreated (no insult; 100% viability) and treated (glutamic acid; 5% viability) controls. At least four wells were used in assays, repeated a minimum of three to four times independently.

### Intracellular calcium kinetics

Intracellular calcium influx was monitored as previously described [8]. Briefly, primary cortical neuronal cultures were loaded with the fluorescent calcium ion indicator Fura-2 AM (5 μM; Sigma Aldrich) in 50 μL MEM/B27, 0.1% pluronic F-127 (Sigma Aldrich), for 20 min at 37 °C in the CO_2_ incubator. Fura-2 AM solution was removed from wells, replaced with 50 μL MEM/B27 containing peptides (R18 and R18D; 0.2, 2 and 5 μM) or NMDA and AMPA receptor blockers (MK801/CNQX; 5 μM/5 μM; Tocris Bioscience, Bristol, United Kingdom) and incubated for 10 min at 37 °C in the CO_2_ incubator. Control cultures received 50 μL of MEM/B27 only. After the 10 min incubation period, media in wells was replaced with 50 μL of balanced salt solution (BSS: mM: 116 NaCl, 5.4 KCl, 1.8 CaCl_2_, 0.8 MgSO_4_, 1 NaH_2_PO_4_; pH 7.2) and wells transferred to a spectrophotometer (BMG Labtec, CLARIOstar, Mornington, Australia) while maintaining temperature at 37 °C. Fifty microliters of BSS containing glutamic acid (200 μM; 100 μM final concentration) was added to wells, and every 5 s, starting 30 s before and for 90 s after glutamic acid addition, spectrophotometer measurements (excitation: 355 nm/emission 495 nm) were recorded. Experiments were performed in triplicate. Area under the curve (AUC) for calcium kinetics data was calculated by trapezoidal approximation of the AUC using fluorescent kinetic data obtained after the addition of glutamic acid to wells.

### Statistical analysis

The number of animals used in each experiment was justified by statistical power calculation based on a previous study [60]. Group sizes have been calculated based on a predicted treatment effect of 40%, at a power level of 80–90% and an alpha level of 0.05. Mean percentage infarct volume, behavioural measurements weight gain and neuronal viability data were analysed by analysis of variance (ANOVA), followed by post-hoc Fisher’s PLSD test, with *P* < 0.05 values considered statistically significant. All descriptive statistics are presented in Table 3 and Additional file 1: Table S1, Additional file 2: Table S2 and Additional file 3: Table S3.

## Results

### Infarct volume measurements

Data on infarct volume and representative TTC stained coronal brain slices are presented in Fig. 1a and b. When compared to the saline vehicle control (hereafter referred to as saline), both R18 and R18D significantly reduced infarct volume at all doses examined (30, 100, 300 and 1000 nmol/kg) by between 25.27 and 43.80% (*P* = 0.045 and < 0.001, respectively; refer to Table 3 for descriptive statistics). By comparison, the JNKI-1-TATD peptide (1000 nmol/kg) reduced infarct volume by 25.27% (*P* = 0.073). No single R18, R18D or JNKI-1-TATD dose was significantly more effective than any other dose. To assess infarct development within 2 mm coronal slices, rostral to caudal topographic analysis of infarcts revealed that R18, R18D and JNKI-1-TATD at all doses significantly reduced brain injury in one or more coronal slices 1 to 6 (Fig. 1c; refer to Additional file 1: Table S1 for descriptive statistics).

### Behavioural outcomes, weight gain and gender analysis

**Fig. 2:**
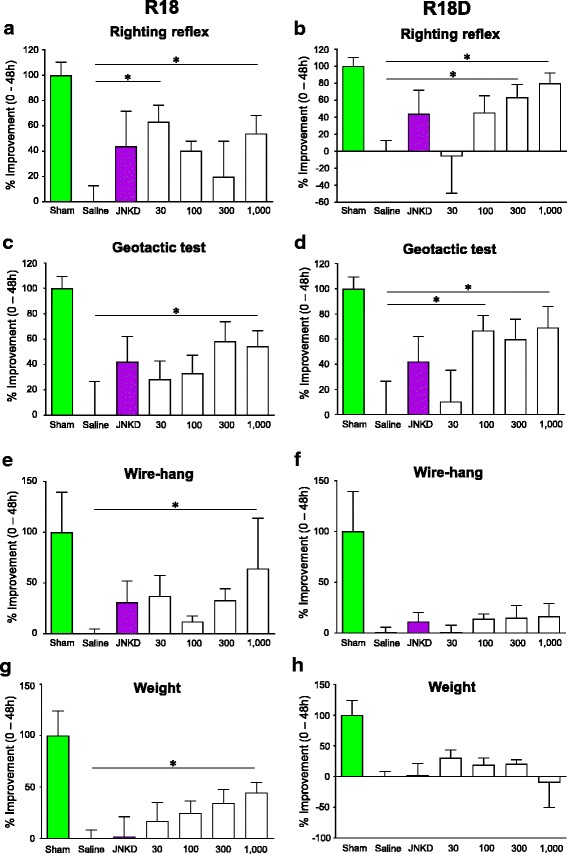
Behavioural measurements using righting reflex, negative geotactic response, wire-hang test and weight gain 48 h after HI. Treatments were administered intraperitoneally (saline, R18, R18D or JNKI-1-TATD; doses in nmol/kg). The sham procedure group was assessed 48 h following sham-surgery. **a** R18 righting reflex percentage improvement from 0 to 48 h. **b** R18D righting reflex percentage improvement from 0 to 48 h. **c** R18 negative geotactic response percentage improvement from 0 to 48 h. **d** R18D negative geotactic response percentage improvement from 0 to 48 h. **e** R18 wire-hang percentage improvement from 0 to 48 h. **f** R18D wire-hang percentage improvement from 0 to 48 h. **g** R18 weight percentage improvement from 0 to 48 h. **h** R18D weight percentage improvement from 0 to 48 h. Values are mean ± SE. *P < 0.05 when compared to saline. JNKD = JNKI-1-TATD

Data on behavioural outcomes and weight gain is presented in Fig. 2a–h and descriptive statistics presented in Additional file 2: Table S2. All data is transformed to demonstrate the percentage improvement from baseline for each treatment group, 48 h following HI. The sham procedure is taken as equivalent to 100% improvement and saline control to 0% improvement. The results of the righting reflex test demonstrated that R18 (30 and 1000 nmol/kg) and R18D (300 and 1000 nmol/kg) significantly improved the righting reflex time by between 53.95% and 79.88% (*P* = 0.008 and 0.007), respectively, when compared to saline. In comparison, animals treated with JNKI-1-TATD (1000 nmol/kg) showed no statistically significant improvement, although there was a trend towards improvement (43.87%; *P* = 0.063).

Analysis of the negative geotactic response test revealed that R18 (1000 nmol/kg) and R18D (100 and 1000 nmol/kg) significantly improved the time taken for animals to rotate 150° up the 45° slope when compared to saline by between 54.44% and 69.17% (*P* = 0.049 and 0.036), respectively. All other doses improved the negative geotactic response time by between 10.40% and 59.87% (*P* = 0.738 and 0.057), respectively. In comparison, JNKI-1-TATD (1000 nmol/kg) resulted in no statistically significant improvement, although there was a trend towards improvement (42.18%; *P* = 0.187).

For the wire-hang test, only R18 at a dose of 1000 nmol/kg resulted in a statistically significant improvement in the time animals held onto the wire, increasing hang-time by 64.29% (*P* = 0.033) when compared to saline. All other doses improved the wire hang time by between 0.59% and 37.26% (*P* = 0.997 and 0.227), respectively. By comparison, JNKI-1-TATD (1000 nmol/kg) resulted in no statistically significant improvement in the wire-hang time (30.93%; *P* = 0.374).

Analysis of weight gain over 48 h revealed that R18 at a dose of 1000 nmol/kg was the only treatment to significantly improve weight gain (44.59%; *P* = 0.009) when compared to saline (see Additional file 3: Table S3 for descriptive statistics), while all other doses of R18 and R18D demonstrated an increase in weight by between 16.83% and 34.49% (*P =* 0.327 and 0.065), respectively. In comparison, JNKI-1-TATD (1000 nmol/kg) treated animals demonstrated a gain in weight (1.94%; *P* = 0.935). The only dose to demonstrate a loss of weight was R18D at 1000 nmol/kg (−10.01%; *P* = 0.659).

Analysis of R18 and R18D neuroprotective efficacy in male and female animals revealed a significant reduction in total infarct volume in both sexes when compared to male and female saline treated animals (Additional file 4: Figure S1; note: for analysis the different doses of R18 and R18D were combined). While intra-group comparisons demonstrated no significant differences in total infarct volumes between male and female animals treated with R18, R18D or saline; there was a trend towards reduced infarcts in female animals treated with R18 and R18D (Additional file 4: Figure S1).

### In vitro neuroprotective efficacy and calcium kinetics following glutamic acid excitotoxicity

**Fig. 3:**
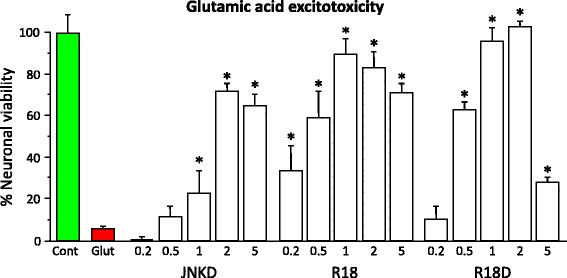
Glutamic acid excitotoxicity model; R18, R18D and JNKI-1-TATD dose response study. Peptides present in neuronal cultures 10 min before and during 5 min glutamic acid exposure. Neuronal viability measured 24 h after glutamic acid exposure. Concentration of peptide in μmol/L. MTS data were expressed as percentage neuronal viability with no insult (100% viability) and glutamic acid control (5% viability). Values are mean ± SE; *n* = 4; *P < 0.05 when compared to no glutamic acid control. Cont = no treatment control. Glut = glutamic acid control. JNKD = JNKI-1-TATD

To provide an insight into the potential mechanism of action of R18 and R18D, in vitro neuroprotective efficacy and excitotoxic neuronal calcium influx kinetics were assessed. In line with previous findings from our laboratory R18 and JNKI-1-TATD [7, 10, 11], as well as R18D displayed dose-dependent neuroprotection following excitotoxicity (Fig. 3). For example, R18 and R18D increased neuronal survival from 5% for the glutamic acid control to 89% and 83% and 96% and 100% at 1 μM and 2 μM, respectively (Fig. 3). By comparison, JNKI-1-TATD increased neuronal survival to 23 and 71% at 1 μM and 2 μM, respectively. As we have previously observed, higher peptide concentrations (i.e. 5 μM) can have a negative impact on neuronal survival [8], this was particularly evident for R18D, a fact which most likely reflects the higher stability of D-isoform peptides in the closed neuronal culture system [9].

**Fig. 4:**
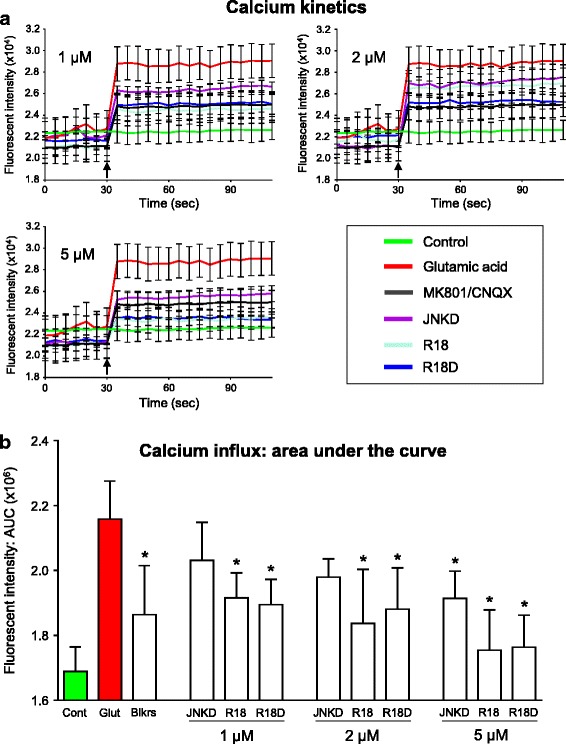
Intracellular calcium assessment using Fura-2 AM after glutamic acid exposure in primary neuronal cultures. **a** Fluorescent Fura-2 AM tracers; fluorescent intensity (FI) of neuronal cultures 30 s before and after the addition (arrow) of glutamic acid (100 μM final concentration). Peptides (1, 2 and 5 μM) or glutamate receptor blockers (MK801/CNQX; 5 μM/5 μM) were added to neuronal cultures for 10 min and removed (time = 0) before glutamic acid addition (time = 30 s). Values are mean ± SE; *n =* 3. **b** Trapezoidal area under the curve (AUC) approximation of calcium kinetic tracers. AUC is determined at 35 s (point after glutamic acid addition. Values are mean ± SE; *n* = 3; *P < 0.05 when compared to glutamic acid control. Cont = no treatment control. Glut = glutamic acid control. Blkrs = glutamate receptor blockers (MK801/CNQX; 5 μM/5 μM). JNKD = JNKI-1-TATD

Again, in line with our previous findings, R18 and JNKI-1-TATD [7, 10, 11], as well as R18D, reduced glutamic acid induced neuronal intracellular calcium influx in a dose dependent manner (Fig. 4). In addition, the calcium influx data for R18, R18D and JNKI-1-TATD correlated with their neuroprotective effectiveness following glutamic acid excitotoxicity, with JNKI-1-TATD displaying the least neuronal intracellular calcium influx inhibitory effects.

## Discussion

The present study extends our previous findings examining the neuroprotective effectiveness of CARPs in in vitro and animal stroke injury models [7–11, 13–16]. In doing so, the data provides compelling evidence for the neuroprotective action of poly-arginine peptides R18 and R18D, as well as the JNKI-1-TATD peptide, which is also a CARP, in a modified Rice-Vannucci model of HIE. The modified Rice-Vannucci model used in this study leads to the generation of a more reproducible infarct, accompanied with a greater severity of brain injury [60]. The original and modified Rice-Vannucci models are likely to mimic the clinical development of a pattern of brain injury, whereby white matter and cerebral cortex, supplied by the middle cerebral and posterior cerebral arteries, are affected following prolonged birth asphyxia [62]. Importantly, the neuroprotective actions of R18 and R18D, as measured in terms of infarct volume, was associated with improved behavioural outcomes at a number of the peptide doses with respect to the righting reflex, negative geotactic response and wire-hang tests, while JNKI-1-TATD only demonstrated trends towards improvement in the righting reflex and negative geotactic response tests. In addition, unlike JNKI-1-TATD, some doses of R18 and R18D improved weight gain following HI. This is another indicator for improved behavioural outcomes as it is likely to reflect the animals’ increased motor capacity to search and feed from the dam, as well as potential reduced weight loss due to increases in respiratory demand, metabolic rate and cachexia [63]. Furthermore, treatment with R18 and R18D was effective at reducing total infarct volume in both male and female animals following HI.

It is noteworthy that, the R18 and R18D were highly effective at reducing infarct volume at all doses examined without displaying a typical dose response effect. The efficacy of both R18 and R18D over a wide dose range (30–1000 nmol/kg) is highly significant, which ultimately could translate to the peptides being effective at low doses when used clinically following HIE. The effectiveness of JNKI-1-TATD (also referred to as XG-102) in a wide dose range with IP administration has also been demonstrated previously following transient MCAO in adult mice [64] and following permanent MCAO in P14 rats [65].

In the present study, R18 and R18D appeared more effective than JNKI-1-TATD across all the in vivo (infarct volume and behavioural outcomes) and in vitro (neuroprotection and neuronal intracellular calcium influx inhibition) outcomes examined. The lower efficacy of JNKI-1-TATD may reflect its lower arginine content (R = 9) and cationic charge (+ 12) compared to R18 and R18D (R = 18, charge = + 18). On this point, our previous studies have confirmed that CARP neuroprotective efficacy and neuronal excitotoxic influx inhibition increases with increasing peptide arginine content and peptide positive charge [8, 9]. It is also noteworthy that in the present study, no obvious overall differences were uncovered between the R18 peptide and its R18D enantiomer.

As mentioned above, the only currently available treatment known to improve neurological outcomes following HIE is moderate hypothermia (32–34 °C for 72 h). As such there is an urgent need for the development of an effective pharmacological neuroprotective agent that can be readily administered following HIE and that is compatible and synergistic with hypothermia. Therefore, it will be important in future studies to assess whether R18 or R18D treatment is compatible with hypothermia, and whether the combined therapies have an additional effect in reducing brain injury and improving behavioural outcomes. In addition, whilst a 48 h endpoint is commonly used in initial neuroprotective and pathophysiological investigations, it is important that the effectiveness of R18 and R18D at more extended time-points should also be evaluated. We also recognise that the IP route for peptide administration may not be ideally suited to the clinical situation, and that an intravenous (IV) route would be preferable. Furthermore, due to the potential differences in blood pharmacokinetics following IP or IV administration of R18 and R18D, at present it is not known how the different delivery routes would impact on the neuroprotective efficacy of the peptides. To this end, further assessment of R18 and R18D when administered by the IV route could be more appropriately carried out in a large animal model of HI (i.e. piglet or lamb).

## Additional files

Additional file 1: Table S1. Cerebral infarct. Percentage of topographical coronal slices. (DOCX 16 kb)

Additional file 2: Table S2. Behavioural assessment. (DOCX 17 kb)

Additional file 3: Table S3. Weight gain 48 h after hypoxia-ischaemia. (DOCX 16 kb)

Additional file 4: Figure S1. Gender comparison of infarct volume. (PPTX 59 kb)

## Abbreviations

ANOVA: Analysis of variance
AUC: Area under the curve
CARPs: Cationic arginine rich peptides
HI: Hypoxia-ischaemia
HIE: Hypoxic ischaemic encephalopathy
HIF1α: Hypoxia-inducible factor 1-α
IP: Intraperitoneal
IV: Intravenous
MCAO: Middle cerebral artery occlusion
MEM: Minimum essential medium
MMPs: Matrix metalloproteinases
MTS: 3-(4,5,dimethyliazol-2-yl)-5-(3-carboxymethoxy-phenyl)-2-(4-sulfophenyl)-2H–tetrazolium salt
NCX3: Sodium calcium exchanger 3
NFκB: Nuclear factor kappa-light-chain enhancer of B cells
NMDA: N-methyl-D-aspartic acid
R12: Poly-arginine-12
R18: Poly-arginine-18
R18D: Poly-arginine-18 D-enantiomer
R9: Poly-arginine-9
ROS: Reactive oxygen species
TTC: 2,3,5-triphenyltetrazolium chloride
