# Supplementary material for: Poly-arginine R18 and R18D (D-enantiomer) peptides reduce infarct volume and improves behavioural outcomes following perinatal hypoxic-ischaemic encephalopathy in the P7 rat
Source: Mol Brain. 2018 Feb 9;11:8. doi: 10.1186/s13041-018-0352-0 (PMC5810179; doi:10.1186/s13041-018-0352-0)
Supplement: Supplementary file 1 — Cerebral infarct. Percentage of topographical coronal slices. (DOCX 16 kb) [file 13041_2018_352_MOESM1_ESM.docx]

**Additional file 1: Table S1.** Cerebral infarct. Percentage of topographical coronal slices.

| **Treatment** | **Slice** | **N** | **Mean (%)** | **SE**  **(%)** | **Percentage reduction** | ***P*** |
| --- | --- | --- | --- | --- | --- | --- |
| Saline | 1 | 19 | 0.683 | 0.148 | - | - |
|  | 2 |  | 2.392 | 0.290 | - | - |
|  | 3 |  | 3.858 | 0.387 | - | - |
|  | 4 |  | 4.124 | 0.358 | - | - |
|  | 5 |  | 4.081 | 0.269 | - | - |
|  | 6 |  | 2.860 | 0.272 | - | - |
| JNKD 1,000 | 1 | 7 | 0.063 | 0.041 | 90.65 | **0.031** |
|  | 2 |  | 1.152 | 0.345 | 51.83 | **0.008** |
|  | 3 |  | 2.973 | 0.805 | 22.93 | 0.211 |
|  | 4 |  | 3.529 | 0.620 | 14.42 | 0.376 |
|  | 5 |  | 3.797 | 0.777 | 6.95 | 0.671 |
|  | 6 |  | 2.170 | 0.536 | 24.12 | 0.200 |
| R18 30 | 1 | 10 | 0.233 | 0.107 | 65.90 | 0.074 |
|  | 2 |  | 1.327 | 0.301 | 44.52 | **0.010** |
|  | 3 |  | 2.243 | 0.363 | 41.86 | **0.011** |
|  | 4 |  | 3.034 | 0.522 | 26.43 | 0.069 |
|  | 5 |  | 2.665 | 0.359 | 34.69 | **0.019** |
|  | 6 |  | 1.732 | 0.182 | 39.44 | **0.030** |
| R18 100 | 1 | 10 | 0.374 | 0.103 | 45.29 | 0.216 |
|  | 2 |  | 1.488 | 0.369 | 37.79 | 0.227 |
|  | 3 |  | 2.514 | 0.574 | 34.83 | **0.033** |
|  | 4 |  | 2.747 | 0.374 | 33.38 | **0.022** |
|  | 5 |  | 2.998 | 0.418 | 26.53 | 0.071 |
|  | 6 |  | 1.852 | 0.309 | 35.24 | 0.051 |
| R18 300 | 1 | 8 | 0.623 | 0.500 | 8.76 | 0.823 |
|  | 2 |  | 1.015 | 0.349 | 57.56 | **0.002** |
|  | 3 |  | 1.747 | 0.344 | 54.71 | **0.002** |
|  | 4 |  | 2.729 | 0.460 | 33.82 | **0.032** |
|  | 5 |  | 3.057 | 0.502 | 25.09 | 0.112 |
|  | 6 |  | 2.606 | 0.535 | 8.88 | 0.644 |
| R18 1,000 | 1 | 11 | 0.067 | 0.038 | 90.07 | **0.013** |
|  | 2 |  | 0.409 | 0.131 | 82.90 | **<0.0001** |
|  | 3 |  | 1.739 | 0.436 | 54.92 | **<0.001** |
|  | 4 |  | 2.541 | 0.476 | 38.38 | **0.007** |
|  | 5 |  | 3.217 | 0.621 | 21.17 | 0.135 |
|  | 6 |  | 2.275 | 0.559 | 20.45 | 0.239 |

**Supplementary Table 1 continued.** Cerebral infarct. Percentage of topographical coronal slices.

| R18D 30 | 1 | 9 | 0.383 | 0.142 | 43.86 | 0.122 |
| --- | --- | --- | --- | --- | --- | --- |
|  | 2 |  | 1.351 | 0.309 | 43.52 | **0.014** |
|  | 3 |  | 3.237 | 0.604 | 16.09 | 0.334 |
|  | 4 |  | 3.263 | 0.575 | 20.87 | 0.130 |
|  | 5 |  | 2.950 | 0.458 | 27.71 | **0.028** |
|  | 6 |  | 2.634 | 0.256 | 7.90 | 0.591 |
| R18D 100 | 1 | 9 | 0.308 | 0.128 | 54.86 | 0.054 |
|  | 2 |  | 1.135 | 0.260 | 52.55 | **0.003** |
|  | 3 |  | 1.702 | 0.265 | 55.88 | **0.001** |
|  | 4 |  | 2.597 | 0.230 | 37.02 | **0.008** |
|  | 5 |  | 2.580 | 0.300 | 36.78 | **0.004** |
|  | 6 |  | 2.008 | 0.151 | 29.79 | **0.046** |
| R18D 300 | 1 | 9 | 0.347 | 0.106 | 49.12 | 0.084 |
|  | 2 |  | 1.296 | 0.359 | 45.81 | **0.010** |
|  | 3 |  | 2.182 | 0.418 | 43.44 | **0.011** |
|  | 4 |  | 2.791 | 0.333 | 32.32 | **0.021** |
|  | 5 |  | 3.092 | 0.378 | 24.23 | 0.533 |
|  | 6 |  | 2.358 | 0.283 | 17.55 | 0.234 |
| R18D 1,000 | 1 | 8 | 0.428 | 0.145 | 37.38 | 0.204 |
|  | 2 |  | 1.425 | 0.217 | 40.42 | **0.028** |
|  | 3 |  | 2.416 | 0.498 | 37.37 | **0.034** |
|  | 4 |  | 2.757 | 0.424 | 33.14 | **0.023** |
|  | 5 |  | 2.845 | 0.188 | 30.28 | 0.213 |
|  | 6 |  | 1.964 | 0.404 | 31.32 | **0.044** |

N, number of animals; SEM, standard error of mean; *P*, calculated compared to saline vehicle control. All doses are in nmol/kg. Mean and SE expressed as percentage of total slice volume. JNKD = JNKI-1-TATD. Percentage reduction expressed as percentage reduction from corresponding saline slice. All values *P* < 0.05 are bold.
