# Supplementary material for: Poly-arginine R18 and R18D (D-enantiomer) peptides reduce infarct volume and improves behavioural outcomes following perinatal hypoxic-ischaemic encephalopathy in the P7 rat
Source: Mol Brain. 2018 Feb 9;11:8. doi: 10.1186/s13041-018-0352-0 (PMC5810179; doi:10.1186/s13041-018-0352-0)
Supplement: Supplementary file 2 — Behavioural assessment. (DOCX 17 kb) [file 13041_2018_352_MOESM2_ESM.docx]

| **Behavioural**  **assessment** | **Treatment** | **Dose** | **N** | **Mean***  **(%)** | **SE**  **(%)** | ***P*** |
| --- | --- | --- | --- | --- | --- | --- |
| Righting  reflex | Sham | - | 6 | 100 | 10.36 | **<0.001** |
| Righting  reflex | Saline | - | 19 | 0 | 13.41 | - |
| Righting  reflex | JNKD | 1,000 | 7 | 43.87 | 27.69 | 0.063 |
| Righting  reflex | R18 | 30 | 10 | 63.21 | 13.16 | **0.003** |
|  |  | 100 | 10 | 40.20 | 7.70 | 0.054 |
|  |  | 300 | 8 | 19.77 | 28.03 | 0.375 |
|  |  | 1,000 | 11 | 53.95 | 14.22 | **0.008** |
| Righting  reflex | R18D | 30 | 9 | -6.18 | 43.28 | 0.822 |
|  |  | 100 | 9 | 45.33 | 19.81 | 0.104 |
|  |  | 300 | 9 | 63.14 | 15.24 | **0.025** |
|  |  | 1,000 | 8 | 79.88 | 11.97 | **0.007** |
| Geotactic  test | Sham | - | 6 | 100 | 9.30 | **0.004** |
| Geotactic  test | Saline | - | 19 | 0 | 26.59 | - |
| Geotactic  test | JNKD | 1,000 | 7 | 42.18 | 19.85 | 0.187 |
| Geotactic  test | R18 | 30 | 10 | 28.23 | 14.48 | 0.317 |
|  |  | 100 | 10 | 32.95 | 14.46 | 0.243 |
|  |  | 300 | 8 | 58.21 | 15.44 | 0.058 |
|  |  | 1,000 | 11 | 54.44 | 12.11 | **0.049** |
| Geotactic  test | R18D | 30 | 9 | 10.40 | 24.84 | 0.738 |
|  |  | 100 | 9 | 66.71 | 12.06 | **0.035** |
|  |  | 300 | 9 | 59.87 | 15.96 | 0.057 |
|  |  | 1,000 | 8 | 69.17 | 16.83 | **0.036** |

**Additional file 2: Table S2.** Behavioural assessment.

**Supplementary Table 2 continued.** Behavioural assessment.

*Mean percentage improvement from baseline to 48 h after HI. N, number of animals; SE, standard error of mean; *P* calculated compared to saline. All doses are in nmol/kg. Mean and SE expressed as percentage improvement in behavioural assessment from baseline to 48 h after HI. All values *P* < 0.05 are in bold.

| Wire  hang | Sham | - | 6 | 100 | 39.27 | **<0.001** |
| --- | --- | --- | --- | --- | --- | --- |
| Wire  hang | Saline | - | 19 | 0 | 4.91 | - |
| Wire  hang | JNKD | 1,000 | 7 | 30.93 | 21.15 | 0.374 |
| Wire  hang | R18 | 30 | 10 | 37.26 | 20.08 | 0.227 |
|  |  | 100 | 10 | 11.99 | 5.579 | 0.695 |
|  |  | 300 | 8 | 32.99 | 11.25 | 0.320 |
|  |  | 1,000 | 11 | 64.29 | 49.70 | **0.033** |
| Wire  hang | R18D | 30 | 9 | 0.59 | 6.67 | 0.997 |
|  |  | 100 | 9 | 13.9 | 4.80 | 0.410 |
|  |  | 300 | 9 | 14.95 | 12.15 | 0.356 |
|  |  | 1,000 | 8 | 16.36 | 12.7 | 0.330 |
