# Supplementary material for: Poly-arginine R18 and R18D (D-enantiomer) peptides reduce infarct volume and improves behavioural outcomes following perinatal hypoxic-ischaemic encephalopathy in the P7 rat
Source: Mol Brain. 2018 Feb 9;11:8. doi: 10.1186/s13041-018-0352-0 (PMC5810179; doi:10.1186/s13041-018-0352-0)
Supplement: Supplementary file 3 — Weight gain 48 h after hypoxia-ischaemia. (DOCX 16 kb) [file 13041_2018_352_MOESM3_ESM.docx]

| **Treatment** | **Dose** | **N** | **Mean (g)*** | **SE (g)** | **Mean (%)^#^** | **SE (%)** | ***P*** |
| --- | --- | --- | --- | --- | --- | --- | --- |
| Sham | - | 6 | 4.6 | 0.841 | 100 | 23.95 | **<0.001** |
| Saline | - | 19 | 1.089 | 0.295 | 0 | 8.424 | - |
| JNKD | 1,000 | 7 | 1.157 | 0.67 | 1.941 | 19.34 | 0.935 |
| R18 | 30 | 10 | 1.68 | 0.64 | 16.83 | 18.32 | 0.327 |
|  | 100 | 10 | 1.95 | 0.42 | 24.52 | 12.07 | 0.155 |
|  | 300 | 8 | 2.30 | 0.46 | 34.49 | 13.20 | 0.065 |
|  | 1,000 | 11 | 2.65 | 0.35 | 44.59 | 10.17 | **0.009** |
| R18D | 30 | 9 | 2.15 | 0.45 | 30.38 | 12.87 | 0.167 |
|  | 100 | 9 | 1.75 | 0.39 | 18.98 | 11.22 | 0.386 |
|  | 300 | 9 | 1.82 | 0.23 | 20.88 | 6.62 | 0.340 |
|  | 1,000 | 8 | 0.73 | 1.42 | -10.01 | 40.50 | 0.659 |

**Additional file 3: Table S3.** Weight gain 48 hours after hypoxia-ischaemia.

*Mean weight gain from P7 (day of injury) to P9 (48 hour endpoint). ^#^Mean percentage weight gain from baseline to 48 h after HI. N, number of animals; g, gram; SE, standard error of mean; *P* calculated compared to saline. All doses are in nmol/kg. Mean and SE are expressed as gram improvement from baseline to 48 h after HI. All values *P* < 0.05 are in bold.
