# Supplementary material for: Poly-arginine R18 and R18D (D-enantiomer) peptides reduce infarct volume and improves behavioural outcomes following perinatal hypoxic-ischaemic encephalopathy in the P7 rat
Source: Mol Brain. 2018 Feb 9;11:8. doi: 10.1186/s13041-018-0352-0 (PMC5810179; doi:10.1186/s13041-018-0352-0)
Supplement: Supplementary file 4 — Gender comparison of infarct volume. (PPTX 59 kb) [file 13041_2018_352_MOESM4_ESM.pptx]

## Slide 1
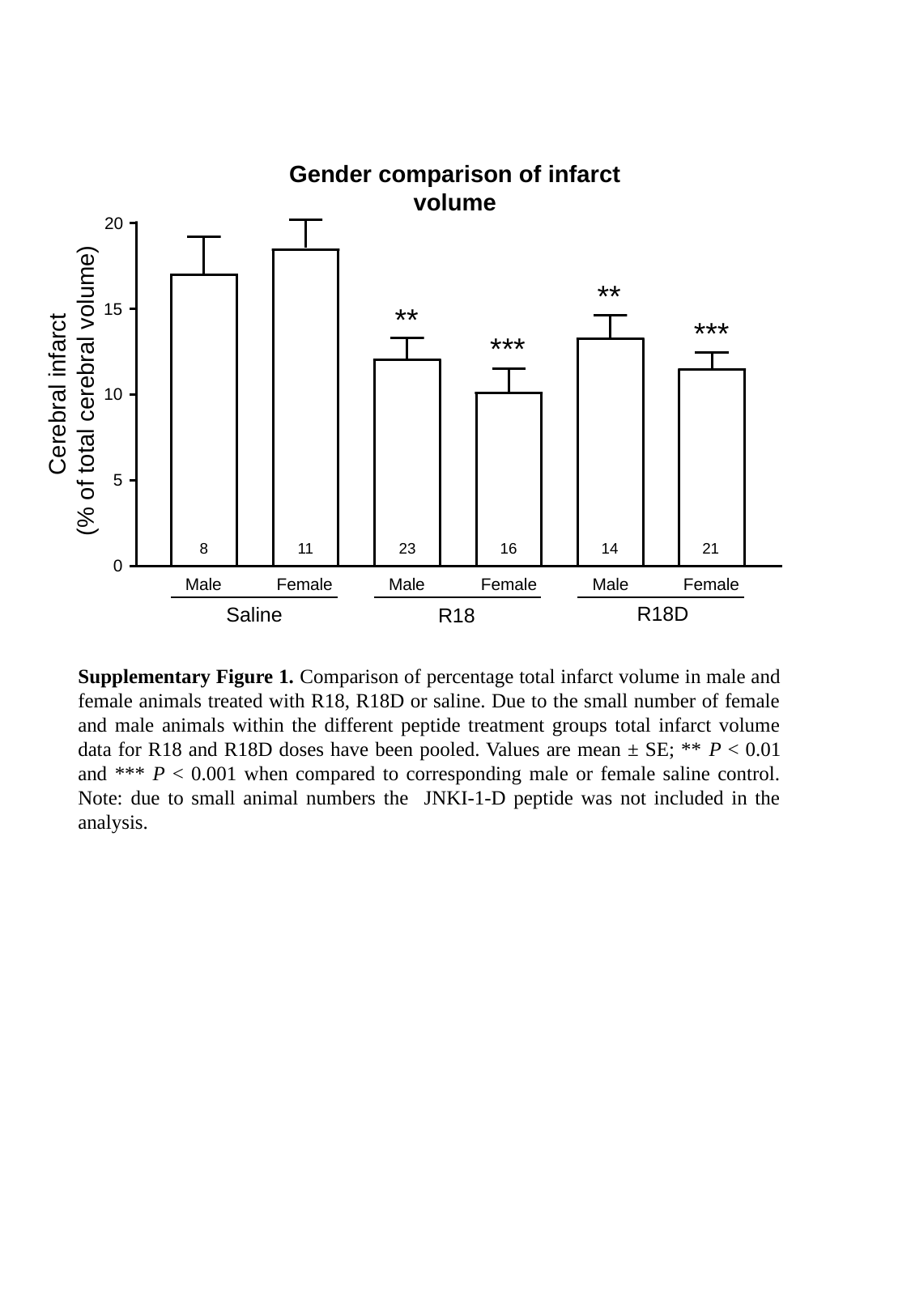

Gender comparison of infarct volume
20
**
15
**
***
***
Cerebral infarct
 (% of total cerebral volume)
10
5
8
21
23
16
14
11
0
Male
Male
Female
Male
Female
Female
R18D
Saline
R18
Supplementary Figure 1. Comparison of percentage total infarct volume in male and female animals treated with R18, R18D or saline. Due to the small number of female and male animals within the different peptide treatment groups total infarct volume data for R18 and R18D doses have been pooled. Values are mean ± SE; ** P < 0.01 and *** P < 0.001 when compared to corresponding male or female saline control. Note: due to small animal numbers the JNKI-1-D peptide was not included in the analysis.
